# Interference of default mode on attention networks in adults with attention‐deficit/hyperactivity disorder and its association with genetic variants and treatment outcomes

**DOI:** 10.1111/cns.14900

**Published:** 2024-08-15

**Authors:** Lu Liu, Di Chen, Fang Huang, Tianye Jia, Wei Cheng, Meirong Pan, Mengjie Zhao, Xuan Bu, Xuhong Liao, Yufeng Wang, Miao Cao, Qiujin Qian, Jianfeng Feng

**Affiliations:** ^1^ Peking University Sixth Hospital/Institute of Mental Health Beijing China; ^2^ NHC Key Laboratory of Mental Health (Peking University) National Clinical Research Center for Mental Disorders (Peking University Sixth Hospital) Beijing China; ^3^ Institute of Science and Technology for Brain‐Inspired Intelligence Fudan University Shanghai China; ^4^ Key Laboratory of Computational Neuroscience and Brain‐Inspired Intelligence, Ministry of Education Fudan University Shanghai China; ^5^ Mental Health Education and Counselling Center Zhejiang University Hangzhou China; ^6^ State Key Laboratory of Cognitive Neuroscience and Learning and IDG/McGovern Institute for Brain Research Beijing Normal University Beijing China; ^7^ School of Systems Science Beijing Normal University Beijing China

**Keywords:** adults with ADHD, cognitive behavioral therapy, connectome analysis, functional connectivity, MAO genotype

## Abstract

**Aims:**

Altered brain functional connectivity has been proposed as the neurobiological underpinnings of attention‐deficit/hyperactivity disorder (ADHD), and the default mode interference hypothesis is one of the most popular neuropsychological models. Here, we explored whether this hypothesis is supported in adults with ADHD and the association with high‐risk genetic variants and treatment outcomes.

**Methods:**

Voxel‐based whole‐brain connectome analysis was conducted on resting‐state functional MRI data from 84 adults with ADHD and 89 healthy controls to identify functional connectivity substrates corresponding to ADHD‐related alterations. The candidate genetic variants and 12‐week cognitive behavioral therapy data were leveraged from the same population to assess these associations.

**Results:**

We detected breakdowns of functional connectivity in the precuneus and left middle temporal gyrus in adults with ADHD, with exact contributions from decreased connectivity within the default mode, dorsal and ventral attention networks, as well as increased connectivity among them with the middle temporal gyrus serving as a crucial ‘bridge’. Additionally, significant associations between the altered functional connectivity and genetic variants in both *MAOA* and *MAOB* were detected. Treatment restored brain function, with the amelioration of connectivity of the middle temporal gyrus, accompanied by improvements in ADHD core symptoms.

**Conclusions:**

These findings support the interference of default mode on attention in adults with ADHD and its association with genetic risk variants and clinical management, providing insights into the underlying pathogenesis of ADHD and potential biomarkers for treatment evaluation.

## Graphical abstract

Our voxel‐based connectome analyses indicated hypoconnectivity within the DMN (the connectivity between the precuneus with other DMN components) and attentional networks (the connectivity between the left middle temporal cluster and other DAN/VAN components) and hyperconnectivity between these networks (the connectivity between the left middle temporal and DMN components) in adults with ADHD, with the middle temporal gyrus as a key ‘bridge’ region.

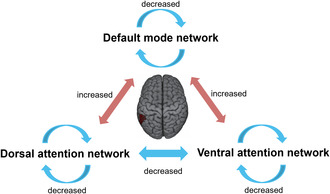

## 1 INTRODUCTION

Attention‐deficit/hyperactivity disorder (ADHD) is a common neurodevelopmental disorder, with a childhood onset (before the age of 12 years) according to the DSM‐5.^1^ In approximately 50% of cases, ADHD persists into adulthood,^2^ although with variable longitudinal patterns,^3^ while the worldwide prevalence of ADHD in adults is approximately 2.5%.^4^ Adults with ADHD have been considered a specific and refined subgroup with poor outcomes, showing stronger family aggregation than those with remission prior to adulthood.^5^, ^6^ Exploration of the underlying pathogenesis of adults with ADHD would substantially enhance our understanding of the etiology of ADHD.

Many famous neuropsychological models have been proposed for ADHD, one of which is the ‘default mode interference hypothesis’ proposed by Sonuga‐Barke and Castellanos.^7^ This hypothesis proposes that the failure of the full and effective transition from the default mode to an active processing mode during cognitive processing causes performance impairments in ADHD. In line with this hypothesis, some studies on adults with ADHD have revealed abnormal activation patterns in the default mode and/or task‐positive networks during cognitive tasks.^8^, ^9^ However, whether the altered functional coordination between the default mode network and the task‐positive networks in adults with ADHD remains during the resting state, a state without specific cognitive demands, requires further elucidation. Several studies have reported abnormal functional connectivity during the resting state in adults with ADHD compared with controls, mainly in the executive control, subcortical, attention, and default mode modules.^10^, ^11^, ^12^ However, the generalization of these findings was limited due to the lack of normal controls,^11^ relatively small sample sizes^10^ and low‐quality MR images (1.5 T MRI).^12^ Thus, further evidence is needed to support the default mode interference hypothesis by exploring the robust alterations in functional organization from the whole‐brain connectome perspective with a large sample size and advanced imaging data. This would provide crucial insights to help us further understand the mechanisms of brain dysfunction in adults with ADHD.

To identify the potential biomarkers of psychopathology, most studies performed group comparisons (ADHD versus healthy controls) to hunt the state‐related brain functional features. From the perspective of predictive validity, the amelioration of these neural indicators through clinical interventions (drug or nondrug treatment) would also promote the elucidation of neural mechanisms. Regarding nondrug treatment, psychotherapy has been suggested to play a role in reducing mental illness symptoms via the ‘top–down’ pathway.^13^ This pathway may directly influence the relevant brain regions which integrating and evaluating relevant information, and then these regions transmitting information down to emotion‐related regions further regulating the release of neurotransmitters. What is more is that the brain functional changes derived from psychotherapy depend on the psychiatric disorder.^13^ Cognitive behavioral therapy (CBT), as a structured psychotherapy method, has been proven to be an effective treatment for multiple mental disorders. For depressive patients, CBT might affect brain regions including anterior cingulate cortex (ACC), posterior cingulate, ventromedial prefrontal cortex/orbitofrontal cortex.^14^ For anxiety‐related disorders, CBT could influence the related regions involving fronto‐insular and fronto‐limbic cortices.^15^ For adults with ADHD, it is still unclear whether CBT could improve the ADHD‐related brain functional alteration, despite few existed reports.^16^

ADHD shows high heritability. Genetic factors that play critical roles in the underlying mechanisms should be key determinants for the occurrence and development of ADHD.^17^ Both candidate genetic studies and genome‐wide association studies have identified several ADHD‐risk genes and variants.^18^, ^19^ Based on the abundant evidence from pharmacological evidence, the dysfunction of monoaminergic system has consistently been suggested to be involved in the etiology of ADHD, and the contribution of genetic variants in the related pathways has been well demonstrated. Monoamine oxidase A and monoamine oxidase B both play a critical role in the metabolism of monoamine neurotransmitters.^20^ Notably, monoamine oxidase A (*MAOA*), located on the human X chromosome, is known as the ADHD distinguishable genotype.^17^ Monoamine oxidase B (*MAOB*), another gene located on the X chromosome, has been showed to be associated with ADHD^21^ and may indicate the persistent status of ADHD.^22^ Based on the ‘gene‐brain‐behavior’ framework, genetic variants might influence the brain development, then the brain structural and/or functional alteration would lead to the cognitive dysfunction and finally brought significant clinical manifestation. That, the imaging features lie intermediate between gene and disorder as endophenotypes.^23^ Imaging genetic studies have indeed suggested that ADHD‐related genetic risk variants might influence clinical symptoms through the mediating effect of brain functional alterations.^24^ The attempt of linking the observed phenotype‐related brain abnormalities with genetic risk variants would enhance our understanding of the molecular genetic underpinnings of the altered connectome organization observed in adults with ADHD from the perspective of construct validity.

To address these gaps, we examined resting‐state functional MRI (fMRI) data from 84 adults with ADHD and 89 healthy controls (HCs) to investigate the alterations of the functional connectome in ADHD and evaluate associations with genotypes and treatment outcomes based on genetic and clinical treatment data in the same population. Specifically, we conducted voxel‐based connectome analysis approaches with multivariate distance matrix regression (MDMR),^25^ seed‐based functional connectivity, and modular analysis methods to comprehensively investigate ADHD‐related functional connectivity alterations. Next, genetic data from a subsample of this population (75 adults with ADHD and 70 HCs) were leveraged to explore the association between the altered connectivity patterns and high‐risk genetic variants. Finally, we explored the potential ‘brain‐treatment’ relationship using data from a subsample of this population (14 adults with ADHD) who received CBT, which is an effective nondrug treatment for adults with ADHD. We hypothesized that in adults with ADHD (i) the interference of the default mode on attention would manifest as disrupted functional connectivity with the corresponding brain networks; (ii) altered connectivity patterns would be significantly associated with high‐risk genetic variants; and (iii) disrupted functional connectivity would be restored after CBT, which may be related to improvements in ADHD core symptoms.

## 2 METHOD

### 2.1 Participants and MRI data acquisition and preprocessing

We obtained imaging data from 84 adults with ADHD and 89 adult healthy controls (HCs) recruited from clinics at Peking University Sixth Hospital/Institute of Mental Health. All ADHD subjects were interviewed by experienced psychiatrists according to the criteria of the Diagnostic and Statistical Manual of Mental Disorders (4th edition; DSM‐IV) using the Conners' Adult ADHD Diagnostic Interview.^26^, ^27^ In addition, structured clinical interviews for DSM‐IV Axis‐I disorders (SCID‐I) were conducted to assess comorbid disorders.^28^ All adults with ADHD had a childhood diagnosis of ADHD. All patients included in this study were unmedicated, that they had never received any psychotropic drugs for ADHD. All included participants (1) were aged 18–45 years; (2) were right‐handed; (3) had no history of severe physical disease; and (4) had a full‐scale intelligence quotient (FSIQ) evaluated using the Wechsler Adult Intelligence Scale‐third edition above 80. Patients with schizophrenia, clinically significant panic disorders, or pervasive developmental disorders were excluded. For HCs, those with any history of psychiatric disorders were excluded. Self‐report data collected with the ADHD Rating Scale‐IV (ADHD RS‐IV) was used to assess the severity of inattentive symptoms, hyperactive/impulsive symptoms, and total ADHD symptoms. The demographic and clinical characteristics are summarized in Table 1. Details of comorbidities are listed in Table S1. This study was approved by the Ethics Committee of Peking University Sixth Hospital/Institute of Mental Health. All participants provided written informed consent.

**TABLE 1:** Demographic and clinical characteristics of baseline and follow‐up studies.

	Baseline study				Follow‐up study		
	ADHD (*n* = 84)	HC (*n* = 89)	*χ* ^2^/*t*	*p*‐value	ADHD (*n* = 14)	*χ* ^2^/*t*	*p*‐value^a^
Male/Female	62/22	66/23	<0.01	0.958	8/6	1.63	0.215
Age (Mean ± SD)	25.80 ± 3.93	26.04 ± 3.63	−0.43	0.668	25.29 ± 3.99	0.45	0.653
Education year (Mean ± SD)	15.88 ± 2.11	17.20 ± 1.97	−4.26	<0.001	16.36 ± 2.68	−0.75	0.454
FSIQ (Mean ± SD)	120.94 ± 7.48	121.83 ± 7.50	−0.78	0.435	120.86 ± 7.85	0.04	0.970
ADHD symptoms (Mean ± SD)							
Inattentive	17.70 ± 3.94				16.93 ± 4.01	0.65	0.518
Hyperactive/Impulsive	10.16 ± 5.11				11.57 ± 6.17	−0.89	0.379
Total	28.14 ± 6.97				28.50 ± 8.96	−0.16	0.872
Comorbidities (*n*, %)	23, 27.38%	—			6, 42.86%	1.38	0.342

Abbreviations: ADHD, attention‐deficit/hyperactivity disorder; FSIQ, full‐scaled intelligence quotient; HC, healthy controls; SD, standard deviation.

^a^ Comparison with total ADHD sample.

Among those subjects recruited for the baseline study, 14 ADHD patients participated in the CBT study (NCT02062411) and had available fMRI data at both baseline and posttreatment; these patients were included in a follow‐up study that tested the predictive validity of the observed alterations in brain functional features. Twelve weeks of group CBT was administered by two trained psychiatrist‐therapists using Safren's validated manual for adult ADHD.^29^ The main elements of this manual included organization and planning, reducing distractibility, adaptive thinking, dealing with procrastination, building helpful relationships, and reviewing. CBT was administered in a 120 min session once per week for 12 weeks with a group of 8 to 12 patients. A detailed description of CBT can be found in our previous work^30^ and also the supplementary materials.

For each participant, the resting‐state fMRI (RS‐fMRI) data were obtained using a 3.0‐Tesla MR system (General Electric; Discovery MR750) in the Center for Neuroimaging at Peking University Sixth Hospital. Specifically, participants in the subgroup from the CBT study were scanned twice at time points just before and after 12 weeks of CBT. The RS‐fMRI images were preprocessed using Statistical Parametric Mapping (SPM8, http://www.fil.ion.ucl.ac.uk/spm) and Data Processing Assistant for Resting‐State fMRI (DPARSF)^31^ following standard procedures. We employed the AAL‐2 template to define the brain's gray matter mask.^32^ Further detail can be found in the Supplementary Materials.

### 2.2 Whole‐brain connectome analysis

To identify the voxelwise biomarkers for adults with ADHD, the preprocessed baseline resting‐state fMRI data (*N*_ADHD_ = 84, *N*_HC_ = 89) were analyzed with the MDMR approach.^25^, ^31^ This method allows for an unbiased, data‐driven approach to determining resources of the changes in functional connectivity. In contrast to most other approaches, MDMR allows a high‐resolution quantification of how a variable of interest (ADHD vs. healthy control groups here) is reflected in the connectivity pattern of individual voxels to the whole brain without parcellating the brain into regions defined a priori. Specifically, for each subject, we calculated a seed‐to‐voxel functional connectivity map within a predefined gray matter mask (*N*_voxel_ = 53,970) for every voxel by estimating the Pearson's correlation coefficient between its time series and all other voxels, resulting in a vector indicating the functional connectivity profile (1×v,v=53,970). Individual differences in the functional connectivity profile for every voxel across every pair of participants were estimated as a metric of distance, as $\sqrt{2\times \left(1-r\right)}$, where the Pearson's correlation coefficient (*r*) is the spatial similarity of the connectivity vector. MDMR was then employed to calculate the relationship between intersubject differences in the connectivity profile and group labels, generating a pseudo‐F statistic for each voxel with age, sex, mean FD, full‐scale IQ, and years of education as covariates. This process was repeated for every single voxel, resulting in a whole brain map of pseudo‐*F* values. The standard permutation flow of 15,000 times was employed to obtain the significance. Suggested by previous literature,^33^ clusters of significant voxels with thresholds defined as *P*_permutation_ < 0.001 and cluster size > 50 voxels were identified.

Notably, this analysis identified a region of interest (ROI), where group labels correlated with functional connectivity, but did not reveal the pattern of connectivity that contributed to the results. Thus, a complimentary seed‐based functional connectivity analysis was employed to explore the connections with specific regions that contributed to these ROIs' abnormalities. To examine the group differences in the seed‐based functional connectivity maps, we employed a general linear model with age, sex, full‐scale IQ, and years of education as covariates. Clusters showing significant group differences (with the following thresholds: voxel‐wise *p* < 0.001; cluster‐corrected *p* < 0.05) were then identified, indicating the target regions.

### 2.3 Analysis of the association of MAOA and MAOB genotypes with ADHD‐related connectivity alterations

Two monoamine oxidase genes, *MAOA* and *MAOB*, from 145 subjects (*N*_ADHD_ = 75, *N*_HC_ = 70) were genotyped. Four SNPs of *MAOA* and eight SNPs of *MAOB* were genotyped using the Sequenom MassARRAY® platform (Sequenom, San Diego, CA, USA). After coding the SNPs, we investigated whether *MAOA* and *MAOB* genotypes could be used for stratification by examining the Pearson correlation coefficient between the two genetic genotypes and the strength of identified abnormal connectivity within each sex with mean FD, age, full‐scale IQ, years of education, and ADHD diagnosis (i.e., ADHD = 1, Control = 0) as the covariates. Additional details are presented in the Supplementary Materials.

### 2.4 Analysis of the association between clinical improvements and ADHD‐related connectivity alterations after CBT

We further examined the association between changes in the identified significantly altered brain connectivity and ADHD total scores after CBT (compared with before treatment) in the subset of patients who underwent treatment. Here, the greater the clinical score reduction after CBT was, the better the behavioral improvement. After regressing out covariates (age, sex, full‐scaled IQ, and years of education) from CBT analysis, a general linear model was employed to explore the relationship. Then, the associations between connectivity changes and ADHD inattention scores and hyperactivity/impulsivity scores were evaluated separately.

For the data recruited in the above analyses, we used the Lilliefors test (*p* > 0.05 indicates a high probability of conforming to a normal distribution) to conduct the normality tests for the identified 42 FCs (i.e., 21 × 2 for both ADHD and controls) at baseline of all individuals (*n* = 84 for ADHD, *n* = 89 for control) and the changes of three symptoms for the follow‐up CBT data (total, inattention, and hyperactivity/impulsivity). We found that the changes of three symptoms are conformed to follow a normal distribution (*p* = 0.253 for total score; *p* = 0.200 for inattention; *p* = 0.500 for hyperactivity/impulsivity), and most FCs (41 of 42, 97.6%) are conformed to follow a normal distribution (*p*‐values >0.05) (Table S2).

## 3 RESULTS

### 3.1 Connectivity discovery with connectome‐wide analysis

**FIGURE 1:**
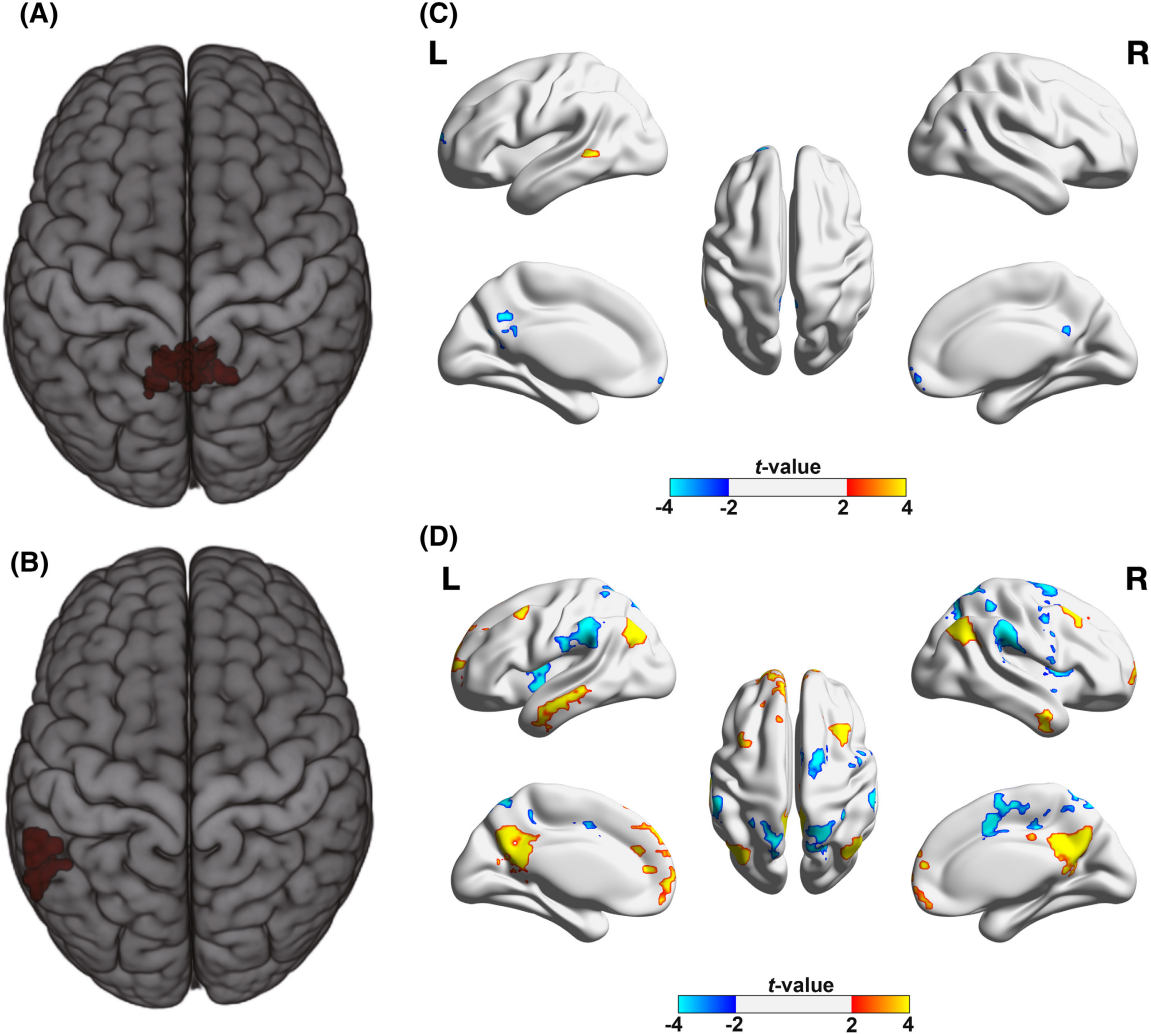
Connectome‐wide connectivity comparisons between adults with ADHD and healthy controls. (A) The MDMR identified cluster showing significant difference in connectivity patterns between adults with ADHD and healthy controls: Left precuneus; (B) The MDMR identified cluster showing significant difference in connectivity patterns between adults with ADHD and healthy controls: Left middle temporal gyrus; (C) Regions whose functional connectivity with precuneus showed significant differences between adults with ADHD and healthy controls (voxel‐wise *P* < 0.001, cluster‐corrected *P* < 0.05); (D) Regions whose functional connectivity with left middle temporal gyrus showed significant differences between adults with ADHD and healthy controls (voxel‐wise *P* < 0.001, cluster‐corrected *P* < 0.05).

MDMR revealed that the differences in functional connectivity patterns between adults with ADHD and healthy controls were concentrated in two clusters: the precuneus and the left middle temporal gyrus (Figure 1A,B). The subsequent connectivity analysis with these two regions as seed ROIs revealed significantly altered connectivity with 21 target ROIs in adults with ADHD compared with HCs (voxel‐wised: *p* < 0.001; cluster‐corrected: *p* < 0.05; Table 2; Figure 1C,D). Notably, these results were retained substantially both in males and females when we further conducted the analyses for each sex separately (Table 2).

**TABLE 2:** Biomarkers of functional connectivity in adults with ADHD. The functional connections showing significant differences between adults with ADHD and HC, which were calculated through the seed‐based functional analysis and general linear model for group comparison. And correlations between the changes of ADHD scores and strength of those connections in adults with ADHD before and after the CBT treatment.

Seed ROI	Target ROI	Biomarkers (ADHD versus HC)						Correlation of changes of ADHD scores and changes of FCs in ADHD								
		Full sample (84 vs. 89)		Male (62 vs. 66)		Female (22 vs. 23)		Total			Inattention			Hyperactivity/Impulsivity		
		*t*	*p*	*t*	*p*	*t*	*p*	*r*	*p*	*p*_*FDR*_	*r*	*p*	*p*_*FDR*_	*r*	*p*	*p*_*FDR*_
Left Middle Temporal Cluster	Precentral_R	−5.97	1.42E‐08	−4.55	1.30E‐05	−4.37	0.0001	0.13	0.6527	0.8901	−0.18	0.5382	0.7688	0.34	0.2416	0.5694
	Supp_Motor_Area_R	−6.46	1.11E‐09	−5.79	5.79E‐08	−3.05	0.0041	−0.39	0.1703	0.3931	−0.42	0.1303	0.4343	−0.30	0.2902	0.6056
	Frontal_Mid_R	6.13	6.25E‐09	5.29	5.63E‐07	3.20	0.0027	0.81	0.0005	0.0068	0.81	0.0005	0.0151	0.69	0.0062	0.0619
	Parietal_Sup_R	−6.26	3.22E‐09	−6.15	1.04E‐08	−1.99	0.0537	−0.01	0.9858	0.9858	0.02	0.9495	0.9495	−0.02	0.9418	0.9495
	Frontal_Mid_L	4.65	6.77E‐06	4.14	6.38E‐05	2.17	0.0364	0.69	0.0063	0.0470	0.49	0.0767	0.3285	0.73	0.0029	0.0438
	Parietal_Sup_L	−6.48	9.81E‐10	−6.40	3.14E‐09	−2.14	0.0388	−0.06	0.8395	0.9796	−0.19	0.5248	0.7688	0.04	0.8940	0.9495
	Temporal_Inf_R	6.10	7.28E‐09	6.64	9.29E‐10	1.63	0.1120	−0.51	0.0611	0.2292	−0.62	0.0178	0.1338	−0.36	0.2082	0.5678
	Temporal_Mid_L	6.24	3.44E‐09	6.53	1.63E‐09	1.93	0.0613	−0.59	0.0248	0.1242	−0.60	0.0224	0.1346	−0.50	0.0684	0.3285
	Frontal_Sup_L	7.13	2.86E‐11	6.67	8.11E‐10	3.08	0.0038	−0.05	0.8569	0.9796	0.09	0.7627	0.8987	−0.15	0.6173	0.8418
	SupraMarginal_L	−5.88	2.18E‐08	−5.37	3.85E‐07	−2.62	0.0125	0.39	0.1658	0.3931	0.46	0.0983	0.3684	0.29	0.3230	0.6056
	Insula_R	−5.90	1.96E‐08	−5.01	1.89E‐06	−3.29	0.0021	0.26	0.3736	0.7006	0.22	0.4508	0.7118	0.25	0.3953	0.6976
	Precuneus	6.41	1.45E‐09	5.44	2.78E‐07	3.42	0.0015	−0.38	0.1834	0.3931	−0.36	0.2024	0.5678	−0.33	0.2467	0.5694
	SupraMarginal_R	−5.67	6.12E‐08	−5.07	1.43E‐06	−2.62	0.0124	0.19	0.5096	0.8177	0.29	0.3132	0.6056	0.09	0.7499	0.8987
	Angular_R	5.88	2.23E‐08	5.80	5.33E‐08	2.02	0.0504	−0.03	0.9143	0.9796	0.05	0.8753	0.9495	−0.08	0.7789	0.8987
	Angular_L	5.74	4.40E‐08	5.30	5.19E‐07	2.33	0.0249	0.18	0.5451	0.8177	0.23	0.4296	0.7118	0.11	0.7007	0.8987
Precuneus Cluster	Frontal_Med_Orb	−5.52	1.29E‐07	−5.78	5.87E‐08	−1.65	0.1072	0.06	0.8507	0.8570	−0.30	0.2927	0.5974	0.30	0.2927	0.5974
	Temporal_Mid_L	5.79	3.48E‐08	4.43	2.11E‐05	4.05	0.0002	−0.30	0.3047	0.8570	−0.26	0.3782	0.5974	−0.28	0.3315	0.5974
	Frontal_Sup_L	−5.57	1.01E‐07	−5.02	1.79E‐06	−2.56	0.0145	0.14	0.6296	0.8570	−0.10	0.7429	0.8104	0.29	0.3143	0.5974
	Calcarine_L	−5.29	3.86E‐07	−4.64	8.86E‐06	−2.82	0.0076	0.05	0.8570	0.8570	−0.04	0.8866	0.8866	0.11	0.7005	0.8104
	Temporal_Mid_R	−4.86	2.69E‐06	−4.30	3.55E‐05	−2.66	0.0114	−0.06	0.8453	0.8570	−0.47	0.0903	0.5974	0.25	0.3983	0.5974
	Precuneus	−5.13	8.01E‐07	−4.34	2.93E‐05	−2.75	0.0090	−0.26	0.3732	0.8570	−0.33	0.2444	0.5974	−0.17	0.5708	0.7611

For the precuneus, 6 target ROIs were identified, among which the PCC/precuneus, the left calcarine cortex, medial frontal gyrus, left superior frontal gyrus, and bilateral middle temporal gyrus all showed positive connectivity with the seed ROI and significantly decreased connectivity in the ADHD group compared with the HC group. For the left middle temporal gyrus, 15 target ROIs could be divided into two groups. One group included the bilateral angular gyrus, bilateral middle frontal gyrus, left superior frontal gyrus, precuneus, and bilateral inferior temporal gyrus; connectivity of the left middle temporal gyrus with these regions was negative in the HC group but positive in the ADHD group. The other group included the right insula, bilateral superior parietal gyrus, bilateral supramarginal gyrus, right precentral gryus, and right SMA; connectivity of the temporal gyrus with these regions was positive in the HC group but negative in the ADHD group. To investigate whether comorbidity affects the stability of the results, we conducted a comorbidity analysis by excluding individuals with comorbidity issues, leaving only the ADHD individuals without comorbidity problems to examine the 21 significant FCs we identified. We found Cohen's *d* values very similar to the original results, demonstrating that our findings are not significantly affected by comorbidity issues (Table S3).

**FIGURE 2:**
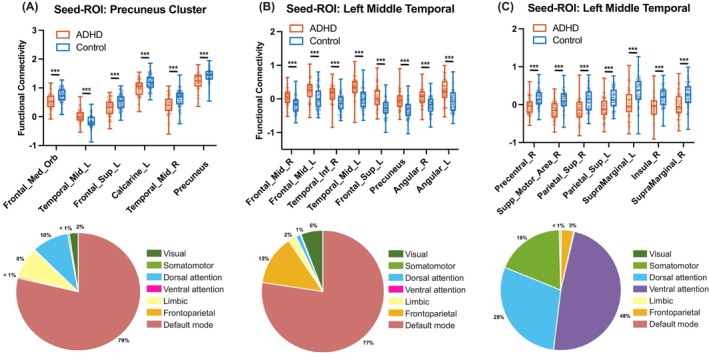
Abnormal functional connections in adults with ADHD compared with healthy controls. (A) Decreased seed‐based functional connectivity of the precuneus in adults with ADHD compared with HCs; (B) Increased seed‐based functional connections of the left middle temporal gyrus in adults with ADHD compared with HCs; (C) Decreased seed‐based functional connections of the left middle temporal gyrus in adults with ADHD compared with HCs. The pie charts in the below row show the distributed percentage of the voxels of target ROIs in every module in corresponding to the results in the upper row. *P < 0.05, **P < 0.01, ***P < 0.001.

To explore the distribution of the abnormal connectivity, we employed a predefined template adapted from Yeo et al.^34^ with 7 networks. For each seed ROI, we calculated the ratio of voxels of themselves and their corresponding target ROIs in every network to the total number of voxels. We found that the precuneus seed was mainly located within the default mode network (94.87%), meanwhile its target ROIs were also mainly located within the same network (78.9%) (Figure 2A; Table S4). In addition, the left middle temporal gyrus seed was located across three networks, including the default mode, frontoparietal, and dorsal attention networks (52.17%, 36.23%, and 10.14%, respectively). Its target ROIs that exhibited abnormal positive connectivity in adults with ADHD were mainly located within the default mode network (77.52%), including the angular gyrus, middle frontal gyrus, precuneus and inferior temporal gyrus, and frontoparietal network (13.22%) (Figure 2B; Table S4). The other target region group, which showed abnormal negative connectivity in adults with ADHD was located within the attention networks (29.29% for the dorsal attention network and 48.36% for the ventral attention network) and somatomotor network (18.47%), including the insula, superior parietal gyrus, supramarginal gyrus, precentral gyrus and SMA (Figure 2C; Table S4).

### 3.2 MAOA and MAOB genotypes related to connectivity alterations in adults with ADHD

**FIGURE 3:**
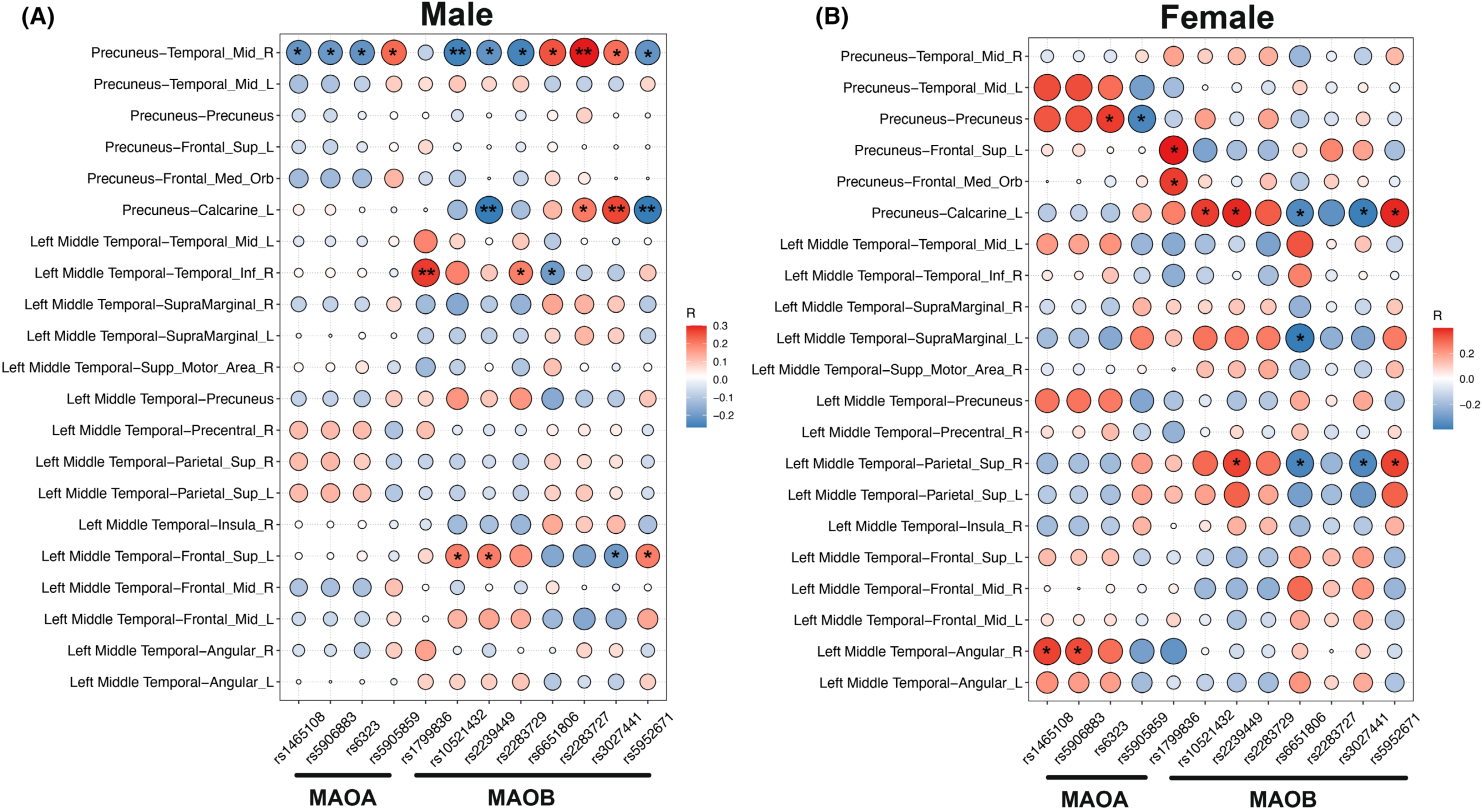
Association between MAO genotypes and the identified ADHD‐altered functional connections. A for males; B for females. R: Right, L: Left. The size of circle is in corresponding to the absolute *r*‐values. Blue for negative values, and red for positive values. *P < 0.05, **P < 0.01, ***P < 0.001.

Significant associations between *MAOA* and *MAOB* genotypes and the strength of identified abnormal connectivity were revealed within each sex, given that *MAOA* and *MAOB* are on the X chromosome. For males (*N*_male_ = 107, Figure 3A), the functional connectivity between the precuneus and right middle temporal gyrus was significantly associated with the genetic variants in both *MAOA* and *MAOB*. For *MAOB* genotypes, significant associations were found with the connectivity between precuneus and left calcarine, the connectivity between left middle temporal gyrus and right inferior temporal gyrus, and the connectivity between left middle temporal gyrus and left dorsolateral superior frontal gyrus (*P*_*uncorrected*_ < 0.05).

For females (*N*_female_ = 38, Figure 3B), the connectivity between the precuneus and precuneus/PCC and the connectivity between the left middle temporal gyrus and right angular were significantly associated with the *MAOA* genotypes (*P*_*uncorrected*_ < 0.05). For *MAOB* genotypes, significant associations were detected with the functional connectivity between the precuneus and left calcarine, the connectivity between the left middle temporal gyrus and right superior parietal gyrus, and the connectivity between the left middle temporal gyrus and left supramarginal gyrus (*P*_*uncorrected*_ < 0.05).

### 3.3 CBT treatment effects on connectivity patterns in adults with ADHD

**FIGURE 4:**
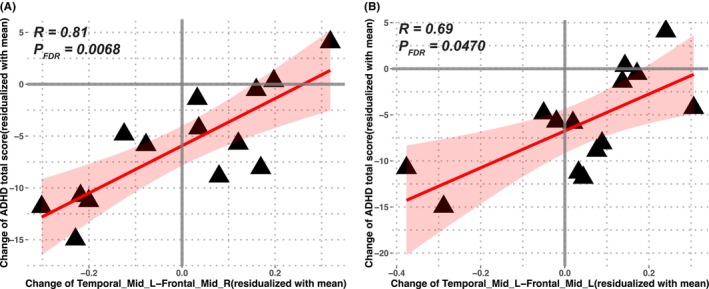
Correlations between the changes in the identified ADHD‐altered functional connections the changes of ADHD total scores after CBT treatment. The positive correlations indicate the larger behavior improvement, the larger connectivity changes, which both have the same direction towards healthy controls. The shaded area is for 95% confidence interval. Temporal‐Mid‐L: Left middle temporal gyrus; Frontal‐Mid‐R: Right middle frontal gyrus; Frontal‐Mid‐L: Left middle frontal gyrus.

For the 14 subjects who participated in the CBT, their ADHD total scores significantly decreased after treatment [(28.50 ± 8.96) versus (20.64 ± 5.34), *t* = 3.92, *p* = 0.002]. The changes in functional connectivity strength between the left middle temporal gyrus and right middle frontal gyrus were significantly correlated with the changes in ADHD scores after CBT (*r* = 0.81, *P*_*FDR*_ = 0.0068 for ADHD total scores, Figure 4A; Table 2; *r* = 0.81, *P*_*FDR*_ = 0.0151 for inattention scores, Table 2). The connectivity between the left middle temporal gyrus and left middle frontal gyrus was marginally correlated with the change in ADHD scores (*r* = 0.69, *P*_*FDR*_ = 0.0470 for ADHD total scores, Figure 4B; Table 2; *r* = 0.73, *P*_*FDR*_ = 0.0438 for hyperactivity/impulsivity scores, Table 2). The positive correlations indicate that the larger connectivity changes, the larger the behavioral improvement was, both in the same direction (i.e., toward that of healthy controls).

## 4 DISCUSSION

We performed voxel‐based connectome analyses to identify brain functional features of adults with ADHD. Briefly, compared with HCs, adults with ADHD showed hypoconnectivity within the DMN (the connectivity between the precuneus with other DMN components) and attentional networks (the connectivity between the left middle temporal cluster and other DAN/VAN components) and hyperconnectivity between these networks (the connectivity between the left middle temporal and DMN components). Further analyses of genetic data and results of a CBT intervention supported the findings in terms of predictive and construct validity. The results of our whole‐brain MDMR analyses provide evidence of the default‐mode interference as an inherit feature in the pathogenesis of adults with ADHD.

The precuneus cluster identified using MDMR included the left precuneus, right precuneus, cingulate gyrus, and posterior cingulate (PCC). The precuneus/PCC was involved in the hub brain regions of the DMN as one part of its posterior components. In our present study, the precuneus seed‐based analyses indicated decreased positive connectivity with other components of the DMN in ADHD patients compared to HCs. This result is consistent with previous reports of atypical default‐mode connectivities in ADHD,^35^, ^36^ indicating altered functional connectivity between posterior and anterior components of the default mode network. In another study,^37^ network homogeneity analyses also showed decreased integrity of the DMN in ADHD, especially regarding connectivity between the precuneus and other components. The altered homogeneity and integration of the DMN in ADHD might influence the switch from the DMN to cognitive modules, which further leads to deficits in cognitive performance. Interestingly, the abnormal patterns of task‐related DMN deactivation and decreased intra‐DMN connectivity could be improved by methylphenidate.^38^, ^39^ In addition to functional impairments, significant alterations in multiple structural indices of the precuneus and PCC have been reported in prior studies of both children and adults with ADHD, including decreased volume, cortical thickness, and surface area.^40^ Future research should examine whether and how these structural and functional abnormalities in DMN regions jointly participate in the pathogenesis of ADHD.

Another ROI identified in the present study was the left middle temporal cluster. Although we found that the left middle temporal gyrus seed was in three networks, including the default mode, frontoparietal, and dorsal attention networks, the correlation analysis revealed that it was generally positively correlated with task positive networks but negatively correlated with the default mode network in HCs. Further seed‐based connectivity analysis indicated different and even opposite directions of signals between adults with ADHD and HCs. Specifically, the negative correlations with default‐mode regions in HCs, such as the precuneus, angular gyrus, and middle frontal cortex, were positive in adults with ADHD, indicating the hyperconnectivity with DMN. Similarly, the positive correlations with regions in the attention networks in HCs, including the insula and superior parietal lobe, were negative in adults with ADHD, indicating hypoconnectivity with task‐relevant regions. Atypical inter‐network connectivity between DMN and cognitive networks has been demonstrated in ADHD patients, which was closely associated with impairments in multiple cognitive domains, including attention and response control.^41^ These findings are consistent with the findings in previous report;^42^ both the hyper‐engaged DMN and hypo‐engaged task‐relevant networks contributed to cognitive impairment. Notably, the altered connectivity between the left temporal and right/left middle frontal clusters was positively correlated with the improvement in ADHD core symptoms after CBT, especially inattention symptoms. This suggests that CBT might improve the ADHD symptoms by influencing the functional connectivity between the temporal lobe and regions in the default‐mode network. In our previous study, the regional homogeneity (ReHo) values of the parahippocampal cluster (including the middle temporal gyrus) in adults with ADHD increased after CBT.^43^ These results support the importance of altered brain functional connectivity of the middle temporal gyrus in the brain mechanisms of ADHD from the perspective of predictive validity. As proposed by Sonuga‐Barke and Castellanos,^7^ default‐mode interference potentially influence the attention variability in subjects with ADHD. The observed DMN‐attention network functional alterations and improvement in inattention symptoms after clinical intervention strongly supported this hypothesis. Attention, referring to both the preparedness for and selection of certain aspects of our physical environment or some ideas in our mind that are stored in memory, its neurobiological basis has been extensively studied by cognitive neuroscientists.^44^ It is now commonly accepted that attention system is composed by a set of independent control networks, which collectively known as the attention networks.^45^ For ADHD, inattention is one of core symptoms, which is more dominant for adults. Based on our present findings, it would be interesting to explore the specified interference of default mode on attention networks when performing the attention task in the future.

Instead of directly calculating internetwork connectivity between the DMN and task‐relevant networks, our present study indicated that the middle temporal gyrus is a key “bridge” region linking the DMN and attention networks, with a potential triggering effect. In general, the middle temporal gyrus is involved in multiple brain networks, including the DMN and attention networks.^46^, ^47^ As demonstrated in the study by Hoogman et al., lower surface area values and cortical thickness of multiple brain regions were found in children with ADHD, including temporal regions.^48^ The delayed structural maturation during childhood might influence the construction and dynamic development of functional connectivity of the temporal gyrus with other brain regions. To further explore and validate the key role of the middle temporal gyrus in the relationship between the DMN and task‐relevant networks, especially attention networks, fusion analysis of both resting‐state and task‐based connectivity could be useful. In addition, the integration of static and dynamic functional connectivity analyses should also be considered.

In addition to predictive validation with the CBT intervention data, we also further leveraged ADHD‐related risk gene (*MAOA*, *MAOB*) data to identify genetic substrates of ADHD‐related alterations in the brain functional connectivity. A strong association was observed between genetic variants and altered intra‐DMN connectivity, such as the association of the connectivity between the precuneus and rMTG with both *MAOA* and *MAOB* variants in males and the association of the connectivity between the precuneus and calcarine with *MAOB* variants in both males and females. Sudre et al. reported that the highest estimated heritability of brain functional connectivity in ADHD occurred in the DMN, which further indicates a significant correlation of functional connectivity with both inattention and hyperactivity/impulsivity symptoms.^49^ A candidate genetic study also suggested that the DMN suppression may potentially mediate the relationship between *DAT1* genetic variants and inattention symptoms in adults with ADHD.^50^ Combined with previous evidence, our present imaging genetic findings support the involvement of the observed brain functional impairments in adults with ADHD from the perspective of construct validity. One point should be treated with caution that the imaging‐genetic correlation was with some difference between males and females. Briefly, the FC between precuneus and right MTG was significantly correlated with virtually all SNPs of *MAOA* and *MAOB* in males, whereas no correlation was found for females. In particular, the correlation was opposite for the FC between precuneus‐right MTG for males and females. In the first step of imaging analyses, the results indicated that the ADHD‐related brain functional features remained both in males and females, with the same association orientation for all markers. Hence, we speculate that the above identified sex difference in the imaging genetic analyses should be mainly from the different genetic mechanisms for males and females. That is, for the same genetic variant, the related risk allele might be different between males and females, even that the associated genotype might be reversed. This phenomenon has been reported in previous genetic studies.^20^, ^51^ However, an expanded sample size should be needed to address this interesting issue more definitively.

Some limitations should be considered. First, we have tried our best to explore the potential confounding influence of sex on our results by setting the sex as one of covariates or performing analysis for each sex separately. The recruitment of more subjects in the future could enable us to elucidate the sex differences in brain imaging features more explicitly.^52^ Second, the secondary analysis of subjects at follow‐up had a relatively small sample size. Using the software G*Power (version 3.1),^53^ we conducted the power analyses (*n* = 14, *α* = 0.05) for the significant associations (*P*_FDR_ <0.05) between changes in ADHD scores and changes in FCs and achieved high statistical power (all Power >0.8, Table S5). However, a rigorous treatment‐imaging study design should be considered in the future to validate our primary findings. Thirdly, our findings here for the default mode interference theory are mainly based on phenomenon descriptions and speculative attributions. Further works with causal design should be conducted in the future. Finally, regarding the imaging genetic analysis, the present sample size was not sufficient to detect the minor effect of common genetic variants that the genetic results could not survive strict Bonferroni correction for multiple testing (*P*_corrected_ <1.98E‐04). Since the *MAOA* and *MAOB* genes we measured have strong ADHD‐related hypotheses^17^, ^21^ and the limited sample size, here we reported the original *P*‐values in the genetics section (i.e., associations between 21 FCs and 12 SNPs), which require further validation with additional data in the future. We analyzed only a few genetic variants of two ADHD‐related genes in the present study. In the future, a larger sample size, more genetic variants, and different genetic parameters (i.e., polygenic risk scores) should be considered.

## 5 CONCLUSION

In summary, we conducted a whole‐brain voxel‐based analysis and explored the potential imaging biomarkers of adults with ADHD, which indicated atypical patterns of brain functional connectivity reflecting the interference of the default‐mode in attention. Further treatment effect and genetic analyses supported the validity of these findings from predictive and construct perspectives based on a concept developed with ADHD animal models.^54^ More importantly, the middle temporal gyrus might be a key “bridge” region that links the DMN and attention networks, which could not only provide information regarding ADHD pathogenesis but also important biomarkers for treatment. A more comprehensive exploration of the middle temporal gyrus is needed and highly important.

## AUTHOR CONTRIBUTIONS

Lu Liu, Fang Huang, Qiujin Qian, Miao Cao, and Di Chen contributed conception and design of the study; Lu Liu, Fang Huang, Mengjie Zhao, Meirong Pan performed the data collection. Di Chen, Miao Cao, Tianye Jia, Wei Cheng, Xuan Bu, and Fang Huang performed the statistical analysis. Lu Liu, Di Chen, Fang Huang, Miao Cao, Qiujin Qian, and Yufeng Wang interpreted the results and wrote the manuscript. Jianfeng Feng revised the manuscript critically. All authors contributed to manuscript revision, read, and approved the submitted version. Lu Liu, Di Chen, Fang Huang contributed equally, and Miao Cao, Qiujin Qian, Jianfeng Feng contributed equally.

## CONFLICT OF INTEREST STATEMENT

The authors declare that the research was conducted in the absence of any commercial or financial relationships that could be construed as a potential conflict of interest.

## Supporting information


Data S1.

## Data Availability

The raw data required to reproduce these findings cannot be shared at this time as the data also forms part of an ongoing study.

## References

[1] American Psychiatric Association . Diagnostic and statistical manual of mental disorders (5th ed.). 2013.

[2] Sibley MH , Mitchell JT , Becker SP . Method of adult diagnosis influences estimated persistence of childhood ADHD: a systematic review of longitudinal studies. Lancet Psychiatry. 2016;3(12):1157‐1165. 27745869 10.1016/S2215-0366(16)30190-0

[3] Sibley MH , Arnold LE , Swanson JM , et al. Variable patterns of remission from ADHD in the multimodal treatment study of ADHD. Am J Psychiatry. 2022;179(2):142‐151. 34384227 10.1176/appi.ajp.2021.21010032 PMC8810708

[4] Dobrosavljevic M , Solares C , Cortese S , Andershed H , Larsson H . Prevalence of attention‐deficit/hyperactivity disorder in older adults: a systematic review and meta‐analysis. Neurosci Biobehav Rev. 2020;118:282‐289. 32798966 10.1016/j.neubiorev.2020.07.042

[5] Chen Q , Brikell I , Lichtenstein P , et al. Familial aggregation of attention‐deficit/hyperactivity disorder. J Child Psychol Psychiatry. 2017;58(3):231‐239. 27545745 10.1111/jcpp.12616

[6] Franke B , Faraone SV , Asherson P , et al. The genetics of attention deficit/hyperactivity disorder in adults, a review. Mol Psychiatry. 2012;17(10):960‐987. 22105624 10.1038/mp.2011.138 PMC3449233

[7] Sonuga‐Barke EJ , Castellanos FX . Spontaneous attentional fluctuations in impaired states and pathological conditions: a neurobiological hypothesis. Neurosci Biobehav Rev. 2007;31(7):977‐986. 17445893 10.1016/j.neubiorev.2007.02.005

[8] Mowinckel AM , Alnaes D , Pedersen ML , et al. Increased default‐mode variability is related to reduced task‐performance and is evident in adults with ADHD. Neuroimage Clin. 2017;16:369‐382. 28861338 10.1016/j.nicl.2017.03.008 PMC5568884

[9] Yang Z , Kelly C , Castellanos FX , Leon T , Milham MP , Adler LA . Neural correlates of symptom improvement following stimulant treatment in adults with attention‐deficit/hyperactivity disorder. J Child Adolesc Psychopharmacol. 2016;26(6):527‐536. 27027541 10.1089/cap.2015.0243 PMC4991601

[10] McCarthy H , Skokauskas N , Mulligan A , et al. Attention network Hypoconnectivity with default and affective network Hyperconnectivity in adults diagnosed with attention‐deficit/hyperactivity disorder in childhood. JAMA Psychiatry. 2013;70(12):1329‐1337. 24132732 10.1001/jamapsychiatry.2013.2174

[11] Soros P , Hoxhaj E , Borel P , et al. Hyperactivity/restlessness is associated with increased functional connectivity in adults with ADHD: a dimensional analysis of resting state fMRI. BMC Psychiatry. 2019;19(1):43. 30683074 10.1186/s12888-019-2031-9 PMC6347794

[12] Mostert JC , Shumskaya E , Mennes M , et al. Characterising resting‐state functional connectivity in a large sample of adults with ADHD. Prog Neuro‐Psychopharmacol Biol Psychiatry. 2016;67:82‐91. 10.1016/j.pnpbp.2016.01.011 PMC4788977 26825495

[13] Barsaglini A , Sartori G , Benetti S , Pettersson‐Yeo W , Mechelli A . The effects of psychotherapy on brain function: a systematic and critical review. Prog Neurobiol. 2014;114:1‐14. 24189360 10.1016/j.pneurobio.2013.10.006

[14] Franklin G , Carson AJ , Welch KA . Cognitive behavioural therapy for depression: systematic review of imaging studies. Acta Neuropsychiatr. 2016;28(2):61‐74. 26122039 10.1017/neu.2015.41

[15] Picó‐Pérez M , Fullana MA , Albajes‐Eizagirre A , et al. Neural predictors of cognitive‐behavior therapy outcome in anxiety‐related disorders: a meta‐analysis of task‐based fMRI studies. Psychol Med. 2023;53(8):3387‐3395. 35916600 10.1017/S0033291721005444

[16] Wang X , Cao Q , Wang J , et al. The effects of cognitive‐behavioral therapy on intrinsic functional brain networks in adults with attention‐deficit/hyperactivity disorder. Behav Res Ther. 2016;76:32‐39. 26629933 10.1016/j.brat.2015.11.003

[17] Nymberg C , Jia T , Lubbe S , et al. Neural mechanisms of attention‐deficit/hyperactivity disorder symptoms are stratified by MAOA genotype. Biol Psychiatry. 2013;74(8):607‐614. 23746540 10.1016/j.biopsych.2013.03.027

[18] Faraone SV , Banaschewski T , Coghill D , et al. The world federation of ADHD international consensus Statement: 208 evidence‐based conclusions about the disorder. Neurosci Biobehav Rev. 2021;128:789‐818. 33549739 10.1016/j.neubiorev.2021.01.022 PMC8328933

[19] Demontis D , Walters GB , Athanasiadis G , et al. Genome‐wide analyses of ADHD identify 27 risk loci, refine the genetic architecture and implicate several cognitive domains. Nat Genet. 2023;55(2):198‐208. 36702997 10.1038/s41588-022-01285-8 PMC10914347

[20] Liu L , Guan LL , Chen Y , et al. Association analyses of MAOA in Chinese Han subjects with attention‐deficit/hyperactivity disorder: family‐based association test, case‐control study, and quantitative traits of impulsivity. Am J Med Genet B Neuropsychiatr Genet. 2011;156B(6):737‐748. 21761555 10.1002/ajmg.b.31217

[21] Li J , Wang Y , Hu S , et al. The monoamine oxidase B gene exhibits significant association to ADHD. Am J Med Genet B Neuropsychiatr Genet. 2008;147(3):370‐374. 17918234 10.1002/ajmg.b.30606

[22] Bonvicini C , Faraone SV , Scassellati C . Common and specific genes and peripheral biomarkers in children and adults with attention‐deficit/hyperactivity disorder. World J Biol Psychiatry. 2018;19(2):80‐100. 28097908 10.1080/15622975.2017.1282175 PMC5568996

[23] Wu Z , Yang L , Wang Y . Applying imaging genetics to ADHD: the promises and the challenges. Mol Neurobiol. 2014;50(2):449‐462. 24687870 10.1007/s12035-014-8683-z

[24] Guo X , Liu L , Li T , et al. Inhibition‐directed multimodal imaging fusion patterns in adults with ADHD and its potential underlying “gene‐brain‐cognition” relationship. CNS Neurosci Ther. 2021;27(6):664‐673. 33724699 10.1111/cns.13625 PMC8111492

[25] Shehzad Z , Kelly C , Reiss PT , et al. A multivariate distance‐based analytic framework for connectome‐wide association studies. NeuroImage. 2014;93:74‐94. 24583255 10.1016/j.neuroimage.2014.02.024 PMC4138049

[26] Epstein JN , Johnson DE . Conners' Adult ADHD Diagnostic Interview for DSM‐IV. MultiHealth Systems; 2001.

[27] Qian Q , Li Y , Wang Y , Zhang Y . An exploratory clinical study of attention deficit hyperactivity disorder in young adulthood. Chin J Nerv Ment Dis. 2010;36(2):75‐79.

[28] First M , Spitzer R , Gibbon M . Structured Clinical Interview for DSM‐IV Axis I Disorders‐Patient Edition (SCIDI/P) (Version 2.0). New York State Psychiatric Institute; 1998.

[29] Safren SA , Otto MW , Sprich S , Winett CL , Wilens TE , Biederman J . Cognitive‐behavioral therapy for ADHD in medication‐treated adults with continued symptoms. Behav Res Ther. 2005;43(7):831‐842. 15896281 10.1016/j.brat.2004.07.001

[30] Huang F , Tang YL , Zhao MJ , et al. Cognitive‐behavioral therapy for adult ADHD: a randomized clinical trial in China. J Atten Disord. 2019;23(9):1035‐1046. 28866911 10.1177/1087054717725874

[31] Yan CG , Zang YF . DPARSF: a MATLAB toolbox for “pipeline” data analysis of resting‐state fMRI. Front Syst Neurosci. 2010;4:13. 20577591 10.3389/fnsys.2010.00013 PMC2889691

[32] Rolls ET , Joliot M , Tzourio‐Mazoyer N . Implementation of a new parcellation of the orbitofrontal cortex in the automated anatomical labeling atlas. NeuroImage. 2015;122:1‐5. 26241684 10.1016/j.neuroimage.2015.07.075

[33] Eklund A , Nichols TE , Knutsson H . Cluster failure: why fMRI inferences for spatial extent have inflated false‐positive rates. Proc Natl Acad Sci USA. 2016;113(28):7900‐7905. 27357684 10.1073/pnas.1602413113 PMC4948312

[34] Yeo BT , Krienen FM , Sepulcre J , et al. The organization of the human cerebral cortex estimated by intrinsic functional connectivity. J Neurophysiol. 2011;106(3):1125‐1165. 21653723 10.1152/jn.00338.2011 PMC3174820

[35] Fair DA , Posner J , Nagel BJ , et al. Atypical default network connectivity in youth with attention‐deficit/hyperactivity disorder. Biol Psychiatry. 2010;68(12):1084‐1091. 20728873 10.1016/j.biopsych.2010.07.003 PMC2997893

[36] Castellanos FX , Margulies DS , Kelly C , et al. Cingulate‐precuneus interactions: a new locus of dysfunction in adult attention‐deficit/hyperactivity disorder. Biol Psychiatry. 2008;63(3):332‐337. 17888409 10.1016/j.biopsych.2007.06.025 PMC2745053

[37] Uddin LQ , Kelly AM , Biswal BB , et al. Network homogeneity reveals decreased integrity of default‐mode network in ADHD. J Neurosci Methods. 2008;169(1):249‐254. 18190970 10.1016/j.jneumeth.2007.11.031

[38] Picon FA , Sato JR , Anes M , et al. Methylphenidate alters functional connectivity of default mode network in drug‐naive male adults with ADHD. J Atten Disord. 2020;24(3):447‐455. 30526190 10.1177/1087054718816822

[39] Liddle EB , Hollis C , Batty MJ , et al. Task‐related default mode network modulation and inhibitory control in ADHD: effects of motivation and methylphenidate. J Child Psychol Psychiatry. 2011;52(7):761‐771. 21073458 10.1111/j.1469-7610.2010.02333.x PMC4754961

[40] Firouzabadi FD , Ramezanpour S , Firouzabadi MD , Yousem IJ , Puts NAJ , Yousem DM . Neuroimaging in attention‐deficit/hyperactivity disorder: recent advances. AJR Am J Roentgenol. 2022;218(2):321‐332. 34406053 10.2214/AJR.21.26316

[41] Duffy KA , Rosch KS , Nebel MB , et al. Increased integration between default mode and task‐relevant networks in children with ADHD is associated with impaired response control. Dev Cogn Neurosci. 2021;50:100980. 34252881 10.1016/j.dcn.2021.100980 PMC8278154

[42] Rubia K . Cognitive neuroscience of attention deficit hyperactivity disorder (ADHD) and its clinical translation. Front Hum Neurosci. 2018;12:100. 29651240 10.3389/fnhum.2018.00100 PMC5884954

[43] Cao Q , Wang X , Qu S , et al. Effects of cognitive‐behavioral therapy on regional homogeneity changes in adults with attention‐deficit/hyperactivity disorder. Chin Ment Health J. 2017;31(3):183‐189.

[44] Raz A , Buhle J . Typologies of attentional networks. Nat Rev Neurosci. 2006;7(5):367‐379. 16760917 10.1038/nrn1903

[45] Petersen SE , Posner MI . The attention system of the human brain: 20 years after. Annu Rev Neurosci. 2012;35:73‐89. 22524787 10.1146/annurev-neuro-062111-150525 PMC3413263

[46] Chiang CT , Ouyang CS , Yang RC , Wu RC , Lin LC . Increased temporal lobe Beta activity in boys with attention‐deficit hyperactivity disorder by LORETA analysis. Front Behav Neurosci. 2020;14:85. 32714161 10.3389/fnbeh.2020.00085 PMC7340165

[47] Arrington CN , Malins JG , Winter R , Mencl WE , Pugh KR , Morris R . Examining individual differences in reading and attentional control networks utilizing an oddball fMRI task. Dev Cogn Neurosci. 2019;38:100674. 31252201 10.1016/j.dcn.2019.100674 PMC6969343

[48] Hoogman M , Muetzel R , Guimaraes JP , et al. Brain imaging of the cortex in ADHD: a coordinated analysis of large‐scale clinical and population‐based samples. Am J Psychiatry. 2019;176(7):531‐542. 31014101 10.1176/appi.ajp.2019.18091033 PMC6879185

[49] Sudre G , Choudhuri S , Szekely E , et al. Estimating the heritability of structural and functional brain connectivity in families affected by attention‐deficit/hyperactivity disorder. JAMA Psychiatry. 2017;74(1):76‐84. 27851842 10.1001/jamapsychiatry.2016.3072 PMC7418037

[50] Brown AB , Biederman J , Valera E , et al. Relationship of DAT1 and adult ADHD to task‐positive and task‐negative working memory networks. Psychiatry Res. 2011;193(1):7‐16. 21596533 10.1016/j.pscychresns.2011.01.006 PMC3105199

[51] Liu L , Chen Y , Li H , et al. Association between SYP with attention‐deficit/hyperactivity disorder in Chinese Han subjects: differences among subtypes and genders. Psychiatry Res. 2013;210(1):308‐314. 23726717 10.1016/j.psychres.2013.04.029

[52] Wierenga LM , Doucet GE , Dima D , et al. Greater male than female variability in regional brain structure across the lifespan. Hum Brain Mapp. 2022;43:470‐499. 33044802 10.1002/hbm.25204 PMC8675415

[53] Faul F , Erdfelder E , Lang AG , Buchner A . G*power 3: a flexible statistical power analysis program for the social, behavioral, and biomedical sciences. Behav Res Methods. 2007;39(2):175‐191. 17695343 10.3758/bf03193146

[54] Nestler EJ , Hyman SE . Animal models of neuropsychiatric disorders. Nat Neurosci. 2020;13:1161. 10.1038/nn.2647 PMC3750731 20877280

