# Supplementary material for: Interference of default mode on attention networks in adults with attention‐deficit/hyperactivity disorder and its association with genetic variants and treatment outcomes
Source: CNS Neurosci Ther. 2024 Aug 15;30(8):e14900. doi: 10.1111/cns.14900 (PMC11325164; doi:10.1111/cns.14900)
Supplement: Supplementary file 1 — Data S1. [file CNS-30-e14900-s001.docx]

**Supplementary Information**

**Methods and Materials**

***Procedures of CBT***

The manual of the 12 weeks of group CBT was published and validated in previous study.^1^ This manual was translated into Chinese language and was effective for Chinese patients in reducing core ADHD symptoms, executive function (EF) and impulsivity.^2^ From the first to 12th week, CBT was conducted weekly. All sessions were of 120 min duration and they were conducted in groups, with each group having 8 to 12 patients. The structured CBT sessions had six modules, including *'organization and planning*', '*reducing distractibility*', '*adaptive thinking*', '*dealing with procrastination*', '*building helpful relationship*', and '*review*'. The '*organization and planning*' part included some behavioral time management strategies such as plan list, task decomposition and file organization. The main content of '*reducing distractibility*' was measure attention span and delay distraction by using a timer and environmental management. These previous two parts focused on behavioral training, and the third part as '*adaptive thinking*' was mainly cognitive correction. The '*adaptive thinking*' included detailed explanation of the ADHD cognitive model, identification and debate of negative spontaneous thinking, and exploration of core beliefs. The '*dealing with procrastination*' part was comprehensive application of previous behavioral and cognitive parts in aim of reducing procrastination. The main purpose of '*building helpful relationship*' was obtaining interpersonal support in patients’ daily life. And there was a '*review*' part to summarize and say goodbye to group members. All these CBT sessions were led by two trained psychiatrist-therapists, who had received systematic training of CBT through a 2-year continuous workshop sponsored by the Chinese Psychological Association. There were written and audio records of each therapy session, and the therapists were supervised by a senior psychiatrist and a psychotherapist on weekly basis.

***MRI acquisition and preprocessing***

For each participant, the resting-state fMRI (R-fMRI) data were obtained using a 3.0-Tesla MR system (General Electric; Discovery MR750) in the Center for Neuroimaging at Peking University Sixth Hospital. Specifically, participants in the sub-dataset for CBT study were scanned twice at time points just before and after 12 weeks of CBT. 8-min R-fMRI data were acquired using an echo planar imaging (EPI) sequence with the following parameters: 43 axial slices, slice thickness = 3.2 mm, slice skip = 0 mm, repetition time (TR) = 2000 ms, echo time (TE) = 30 ms, flip angle (FA) = 90°, matrix = 64×64, field of view (FOV) = 220×220 mm^2^, and 240 volumes. High resolution T1-weighted anatomical images were acquired with the following parameters: 180 sagittal slices, slice thickness = 1 mm, slice skip = 0 mm, TR = 6.66 ms, TE = 2.93 ms, TI = 450 ms, FA = 8°, FOV = 256×256 mm^2^, matrix = 256×256.

The R-fMRI images were preprocessed using Statistical Parametric Mapping (SPM8, http://www.fil.ion.ucl.ac.uk/spm) and Data Processing Assistant for Resting-State fMRI (DPARSF).^3^ Briefly, we discarded the first 10 volumes of each participant, and corrected the acquisition time delay through slice timing and head motion through realignment to the first volume. No subject was excluded under a head motion criteria of 3 mm and 3°. The mean framewise displacements (FD)^4^ of all participant were smaller than 0.5 mm. The individual T1-weighted images were coregistered to the mean functional image after motion correction using a linear transformation and were then segmented into gray matter, white matter, and cerebrospinal fluid tissue maps by using a unified segmentation algorithm with SPM’s apriority tissue maps as references.^5^ The resultant gray matter, white matter, and cerebrospinal fluid images were further nonlinearly registered into the MNI space with the information estimated in unified segmentation and then averaged across all subjects to create custom gray matter, white matter, and cerebrospinal fluid templates. We then applied the transformation parameters estimated during unified segmentation to the motion-corrected functional volumes and resampled the transformational functional images to 3 mm isotropic voxels. The normalized functional images further underwent spatial smoothing with a 4 mm full width at half maximum (FWHM) Gaussian kernel and removal of linear trends. Temporal band-pass filtering (0.01–0.1 Hz) was performed and several nuisance variables, including Friston’s 24 head motion parameters,^6^ the averaged signal from white matter, cerebrospinal fluid tissue and global brain, were further removed through multiple linear regression analysis to reduce the effects of non-neuronal signals. The residuals were used for the following connectome analysis within a gray mask. The gray matter mask was generated through thresholding (cutoff = 0.2) the mean gray matter probability map of all subjects (N_voxel_ = 53,970).

***Association analysis between MAOA and MAOB genotypes and ADHD-related connectivity alterations***

Two monoamine oxidase genes, *MAOA* and *MAOB*, from 145 subjects (N_ADHD_ = 75, N_control_ = 70) were genotyped. Four SNPs (rs1465108, rs5906883, rs6323, rs5905859) of *MAOA* and eight SNPs (rs1799836, rs10521432, rs2239449, rs2283729, rs6651806, rs2283727, rs3027441, and rs5952671) of *MAOB* were genotyped using the Sequenom MassARRAY^®^ platform (Sequenom, San Diego, CA, USA). The SNPs were coded as follows: (1) heterozygote coded as ‘1’ (*e.g.* ‘AC’ coded as ‘1’); (2) homozygous coded as ‘2’ or ‘0’ which follows the letter’s order (*e.g.* ‘AA’ & ‘CC’ coded as ‘2’ for ‘AA’; ‘0’ for ‘CC’).

After the calculation of functional connection strength between the seed ROI and corresponding target ROIs through extracting the averaged time course of every ROI, we investigated whether *MAOA* and *MAOB* genotypes could be used for stratification by testing the association between the two genetic genotypes and the strength of identified abnormal connectivity for each sex with mean FD, age, full-scale IQ, years of education and ADHD diagnosis as the covariates.

**Table S1. Detailed information of comorbidities in 84 adults with ADHD**

| **Comorbidities** | **Numbers of ADHD patients (%)** |
| --- | --- |
| **Affective Disorders (%)** | 17 (20.2) |
| Bipolar disorder (%) | 4 (4.8) |
| Major depressive disorder (%) | 12 (14.3) |
| Dysthymia (%) | 3 (3.6) |
| **Anxiety disorders (%)** | 9 (10.7) |
| Panic disorder (%) | 1 (1.2) |
| Social phobia (%) | 4 (4.8) |
| Specific phobia (%) | 1 (1.2) |
| Obsessive compulsive disorder (%) | 1 (1.2) |
| Generalized anxiety disorder (%) | 4 (4.8) |
| Unspecified anxiety disorder (%) | 1 (1.2) |
| **Eating Disorder (%)** | 3 (3.6) |
| [bulimia nervosa](javascript:;) (%) | 3 (3.6) |

**NOTE.** The total numbers of affective disorders and anxiety disorders are not equal to the sum of the following each disorder, because one patient may be with more than one comorbidity.

**Table S2. *P*-values of Lilliefors test for FCs**

| **Seed ROI** | **Target ROI** | **ADHD (n = 84)** | **Control (n = 89)** |
| --- | --- | --- | --- |
| **Left Middle Temporal Cluster** | Precentral_R | 0.500 | 0.188 |
|  | Supp_Motor_Area_R | 0.500 | 0.394 |
|  | Frontal_Mid_R | 0.368 | 0.500 |
|  | Parietal_Sup_R | 0.237 | 0.153 |
|  | Frontal_Mid_L | 0.337 | 0.139 |
|  | Parietal_Sup_L | 0.500 | 0.095 |
|  | Temporal_Inf_R | 0.316 | 0.500 |
|  | Temporal_Mid_L | 0.259 | 0.500 |
|  | Frontal_Sup_L | 0.350 | 0.392 |
|  | SupraMarginal_L | 0.160 | 0.178 |
|  | Insula_R | 0.406 | 0.401 |
|  | Precuneus | 0.120 | 0.500 |
|  | SupraMarginal_R | 0.221 | 0.406 |
|  | Angular_R | 0.500 | 0.500 |
|  | Angular_L | 0.500 | 0.051 |
| **Precuneus Cluster** | Frontal_Med_Orb | 0.500 | 0.500 |
|  | Temporal_Mid_L | 0.466 | 0.171 |
|  | Frontal_Sup_L | 0.481 | 0.274 |
|  | Calcarine_L | 0.062 | 0.500 |
|  | Temporal_Mid_R | 0.500 | 0.002 |
|  | Precuneus | 0.061 | 0.196 |

**Table S3. Result of comorbidity analyses (Cohen's d)**

| **Seed ROI** | **Target ROI** | **ADHD vs. HC** | **ADHD without comorbidity**  **vs. HC** |
| --- | --- | --- | --- |
| **Left Middle Temporal Cluster** | Precentral_R | -0.927 | -0.929 |
|  | Supp_Motor_Area_R | -1.003 | -0.940 |
|  | Frontal_Mid_R | 0.952 | 0.879 |
|  | Parietal_Sup_R | -0.972 | -1.031 |
|  | Frontal_Mid_L | 0.722 | 0.737 |
|  | Parietal_Sup_L | -1.007 | -1.061 |
|  | Temporal_Inf_R | 0.947 | 0.969 |
|  | Temporal_Mid_L | 0.970 | 0.975 |
|  | Frontal_Sup_L | 1.108 | 1.114 |
|  | SupraMarginal_L | -0.913 | -0.871 |
|  | Insula_R | -0.917 | -0.879 |
|  | Precuneus | 0.995 | 1.020 |
|  | SupraMarginal_R | -0.881 | -0.752 |
|  | Angular_R | 0.913 | 0.920 |
|  | Angular_L | 0.891 | 0.911 |
| **Precuneus Cluster** | Frontal_Med_Orb | -0.857 | -0.779 |
|  | Temporal_Mid_L | 0.899 | 0.898 |
|  | Frontal_Sup_L | -0.865 | -0.843 |
|  | Calcarine_L | -0.821 | -0.830 |
|  | Temporal_Mid_R | -0.755 | -0.599 |
|  | Precuneus | -0.797 | -0.634 |

**Table S4.** **Grouping of target ROIs which showed significant differences in the connectivity with seed ROI between adults with ADHD and HC with the Yeo-7 template.**

|  | | **Visual Network** | **Somatomotor Network** | **Dorsal Attention Network** | **Ventral Attention Network** | **Limbic Network** | **Frontoparietal Network** | **Default Mode Network** |
| --- | --- | --- | --- | --- | --- | --- | --- | --- |
| **Left middle temporal gyrus cluster** | Number of voxels (%) | 0 (0) | 1 (1.45) | 7 (10.14) | 0 (0) | 0 (0) | 25 (36.23) | 36 (52.17) |
| Temporal-targeted ROIs with increased connectivity | Number of voxels (%) | 132 (5.82) | 0 (0) | 28 (1.23) | 5 (0.22) | 45 (1.98) | 300 (13.22) | 1759 (77.52) |
| Temporal-targeted ROIs with decreased connectivity | Number of voxels (%) | 7 (0.42) | 309 (18.47) | 490 (29.29) | 809 (48.36) | 0 (0) | 56 (3.35) | 2 (0.12) |
| **Precuneus Cluster** | Number of voxels (%) | 6 (5.13) | 0 (0) | 0 (0) | 0 (0) | 0 (0) | 0 (0) | 111 (94.87) |
| Precuneus-targeted ROIs | Number of voxels (%) | 5 (2.29) | 1 (0.46) | 21 (9.63) | 0 (0) | 18 (8.26) | 1 (0.46) | 172 (78.9) |

**NOTE.** The ratio is calculated as the number of voxels in every network to the total number of voxels of target ROI.

**Table S5. Post-hoc power analysis for the results**

| **Seed ROI** | **Target ROI** | **Correlation of changes of ADHD scores and changes of FCs (n=14)** | | | | | |
| --- | --- | --- | --- | --- | --- | --- | --- |
|  |  | **Total** | | **Inattention** | | **Hyperactivity/Impulsivity** | |
|  |  | ***r*-value** | **Power** | ***r*-value** | **Power** | ***r*-value** | **Power** |
| **Left Middle Temporal** | **Frontal_Mid_R** | 0.81 | 0.97 | 0.81 | 0.97 | / | / |
|  | **Frontal_Mid_L** | 0.69 | 0.83 | / | / | 0.73 | 0.89 |

**References:**

1. Safren SA, Otto MW, Sprich S, Winett CL, Wilens TE, Biederman J. Cognitive-behavioral therapy for ADHD in medication-treated adults with continued symptoms. *Behaviour Research and Therapy* 2005; 43 (7): 831-842.
2. Huang F, Tang YL, Zhao MJ, Wang YF, Pan MR, Wang YF et al. Cognitive-Behavioral Therapy for Adult ADHD: A Randomized Clinical Trial in China. *Journal of Attention Disorders* 2019; 23 (9): 1035-1046.

3. Chao-Gan Y, Yu-Feng Z. DPARSF: A MATLAB Toolbox for "Pipeline" Data Analysis of Resting-State fMRI. *Front Syst Neurosci* 2010; 4: 13.

4. Power JD, Mitra A, Laumann TO, Snyder AZ, Schlaggar BL, Petersen SE. Methods to detect, characterize, and remove motion artifact in resting state fMRI. *Neuroimage* 2014; 84: 320-341.

5. Ashburner J, Friston KJ. Unified segmentation. *Neuroimage* 2005; 26(3):839-851.

6. Friston KJ, Williams S, Howard R, Frackowiak RS, Turner R. Movement-related effects in fMRI time-series. *Magn Reson Med* 1996; 35(3): 346-355.
